# Gens PSD‐95 and GSK‐3β expression improved by hair follicular stem cells‐conditioned medium enhances synaptic transmission and cognitive abilities in the rat model of vascular dementia

**DOI:** 10.1002/brb3.3351

**Published:** 2023-12-31

**Authors:** Mojtaba Ghobadi, Somayeh Akbari, Mahnaz Bayat, Seyed Mostafa Shid Moosavi, Mohammad Saied Salehi, Sareh Pandamooz, Negar Azarpira, Afsoon Afshari, Etrat Hooshmandi, Masoud Haghani

**Affiliations:** ^1^ Department of Physiology Shiraz University of Medical Sciences Shiraz Iran; ^2^ Histomorphometry and Stereology Research Centre Shiraz University of Medical Sciences Shiraz Iran; ^3^ Clinical Neurology Research Centre Shiraz University of Medical Sciences Shiraz Iran; ^4^ Stem Cells Technology Research Center Shiraz University of Medical Sciences Shiraz Iran; ^5^ Shiraz Institute of Stem Cell and Regenerative Medicine Shiraz University of Medical Sciences Shiraz Iran; ^6^ Shiraz Nephro‐Urology Research Center Shiraz University of Medical Sciences Shiraz Iran

**Keywords:** conditioned medium, long‐term potentiation, memory, synaptic transmission, vascular dementia

## Abstract

**Introduction:**

Vascular dementia (VaD) is a common type of dementia. The aim of this study was to investigate the cellular and molecular mechanism of conditioned medium (CM) in VaD.

**Material and methods:**

The rats were divided into four groups of control (*n* = 9), sham‐operation (*n* = 10), VaD with vehicle (*n* = 9), and VaD with CM (*n* = 12) that received CM on days 4, 14, and 24 after 2VO. Before sacrificing the rats, cognitive performance was assessed through the open‐field (OP), passive‐avoidance, and Morris‐water maze. The field‐potential recording was used to investigate basal synaptic transmission (BST) and long‐term potentiation (LTP). Subsequently, the hippocampus was dissected, and real‐time PCR was used to quantify the expression levels of β1‐catenin, insulin‐like growth factor‐1 (IGF‐1), transforming growth factor‐beta (TGF‐β), glycogen synthase kinase‐3β (GSK‐3β), postsynaptic density protein 95 (PSD‐95), and NR2B genes.

**Results:**

The results indicated impaired performance in behavioral tests in 2VO rats, coupled with reductions in BST and LTP induction. The expression levels of β1‐catenin, IGF‐1, PSD‐95, and TGF‐β genes decreased, whereas NR2B and GSK‐3β expression increased. Treatment with CM restores the expression of PSD‐95 and GSK‐3β as well as fear‐memory, spatial learning, and grooming number without a positive effect on memory retrieval, time spent on the periphery and center of OP. The BST recovered upon administration of CM but, the LTP induction was still impaired.

**Conclusion:**

The recovery of BST in VaD rats appears to be the most important outcome of this study which is caused by the improvement of gene expression and leads to the restoration of fear memory.

## Graphical abstract

Vascular dementia (VaD) is a common type of dementia that occurs when there is damage to the blood vessels in the brain. While there is no cure for VaD, treatment focuses on managing underlying conditions and controlling risk factors. In recent years, there has been an increasing interest in the use of conditioned medium (CM) for the treatment of degenerative diseases. Thus, this study aimed to investigate the cellular and molecular mechanism of CM administration in VaD Our data indicated that treatment with CM led to the recovery of basal synaptic transmission and cognitive performance in VaD rats which is probably caused by the improvement of gene expression.

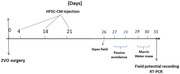

## 1 INTRODUCTION

Vascular dementia (VaD) is the second most prevalent type of age‐related dementia following Alzheimer's disease (AD), characterized by cognitive impairment resulting from vascular abnormalities in the absence of other pathological factors (Kalaria, 2016). The incidence of VaD is swiftly rising among the elderly, affecting approximately 0.98% of individuals aged 71–79 and increasing to 4.09% in those aged 80–89 (Smith, 2017). Various VaD risk factors such as aging, coronary artery disease, middle‐aged hypercholesterolemia, hypertension, obesity, metabolic syndrome, atherosclerosis, smoking, and poor education contribute to oxidative damage in capillary endothelium and vascular dysfunction, resulting in chronic cerebral hypoperfusion (CCH) and subsequent catastrophic lesions (Gao et al., 2013; O'Brien & Thomas, 2015).

Cell therapy is a growing approach that provides strong therapeutic potential for chronic neurodegenerative diseases (Sarti et al., 2002). However, challenges in stem cell transplantation, including cell heterogeneity, ambiguous cell characteristics, immune reactions, low survival, inability to migrate, poor differentiation, and inability to form functional bidirectional synapses with host cells, have been noted (Jayaraman et al., 2013; Lodi et al., 2011). Alternatively, evidence suggests that the regenerative process after cell therapy is primarily attributed to the secretion of trophic factors, such as growth factors, tissue repair factors, various cytokines, and even RNA fragments, rather than the cells themselves (Rusu et al., 2016). Therefore, recently researchers have examined the individual role of different secretory factors, such as secretomes, microvesicles, extracellular vesicles, or exosomes in the culture medium of stem cells (D'Arrigo et al., 2019; Kim et al., 2022). The culture medium containing these factors is called a conditioned medium (CM) (Dowling & Clynes, 2011). In recent years, there has been a growing interest in utilizing CM in regenerative medicine due to its advantages over stem cell use. CM can be artificially produced, packaged, freeze‐dried, and easily transported, and it can be injected repeatedly without the risk of rejection (Pawitan, 2014). Several attempts have been made to understand the beneficial effects of stem cell‐derived CM on dementia (Jiang et al., 2019; Mita et al., 2015; Skok, 2021). Previous studies have reported increased memory performance following the transplantation of hair follicular stem cells (HFSCs) in AD (Esmaeilzade et al., 2014) and VaD models (Akbari et al., 2022). Recently, the HFSC has received attention in the treatment of neurodegenerative diseases due to its potential for autologous transplantation, and it is easily accessibile from a patient's hair follicles (Achilleos & Trainor, 2012). Despite these advantages, the limitations of stem cell transplantation, such as the time‐consuming and expensive nature of obtaining large numbers of cells and low survival rates, have led to focus on CM in treatment (Burst et al., 2010; Choumerianou et al., 2008). So far, HFSC‐CM has shown protective effects in animal models of lung ischemia (Peng et al., 2017) and ischemic stroke (Karimi‐Haghighi et al., 2023).

Previous studies have reported the positive effects from the injection of embryonic stem cells–MSCs and human dental pulp CM into the brain, enhancing hippocampal synaptic transmission, and plasticity in stroke and AD models (Asgari Taei et al., 2021; Mita et al., 2015). Based on the importance of different aspects of synaptic function and structure in the pathophysiology of dementia (Arancio & Chao, 2007), our study aims to investigate the potential protective effects of HFSC‐CM on the VaD model, focusing on memory impairment and synaptic plasticity. As the hippocampus is a key brain area for learning and memory, this study is designed to evaluate synaptic transmission and plasticity at CA3‐CA1 hippocampal excitatory synapses. Additionally, Morris‐water maze (MWM), passive‐avoidance (PA), and open‐field (OP) tests were conducted to assess spatial learning memory, fear memory, and anxiety levels in animals, respectively. We also measured the relative mRNA expression level of the NR2B subunit, a key *N*‐methyl‐d‐aspartate receptor subunit, as well as glycogen synthase kinase‐3β (GSK‐3β), postsynaptic density protein 95 (PSD‐95), and hippocampal mRNA expression level of insulin‐like growth factor‐1 (IGF‐1) and transforming growth factor‐beta (TGF‐β) as neurotrophic factors. These factors play critical roles in neurotransmission regulation, the maintenance of synapses, and hippocampal synaptic plasticity and act as major regulators of synapses maturation and strength (Bradley et al., 2012; Caraci et al., 2015; Coley & Gao, 2018; Gong et al., 2012; Paoletti et al., 2013; Williams et al., 2022).

## 2 MATERIALS AND METHODS

### 2.1 Animals and grouping

In this study, 44 Sprague Dawley male rats (250–300 g, 8–10 weeks old) were used. All animals were housed at standard conditions with controlled temperature (23 ± 1°C), humidity (50% ± 10%), and a 12:12 h light: dark cycle. They had ad libitum access to food and water. The rats were randomly divided into four groups: control (*n* = 10), sham‐operation (*n* = 10), VaD with vehicle (phosphate buffer saline) (VaD + V; *n* = 10), and VaD with CM (VaD + CM; *n* = 14). Four of the rats died intraoperatively and the final grouping changed as follows: the control (*n* = 9), sham‐operation (sham, *n* = 10), VaD + V (*n* = 9), and VaD + CM (*n* = 12).

**FIGURE 1:**
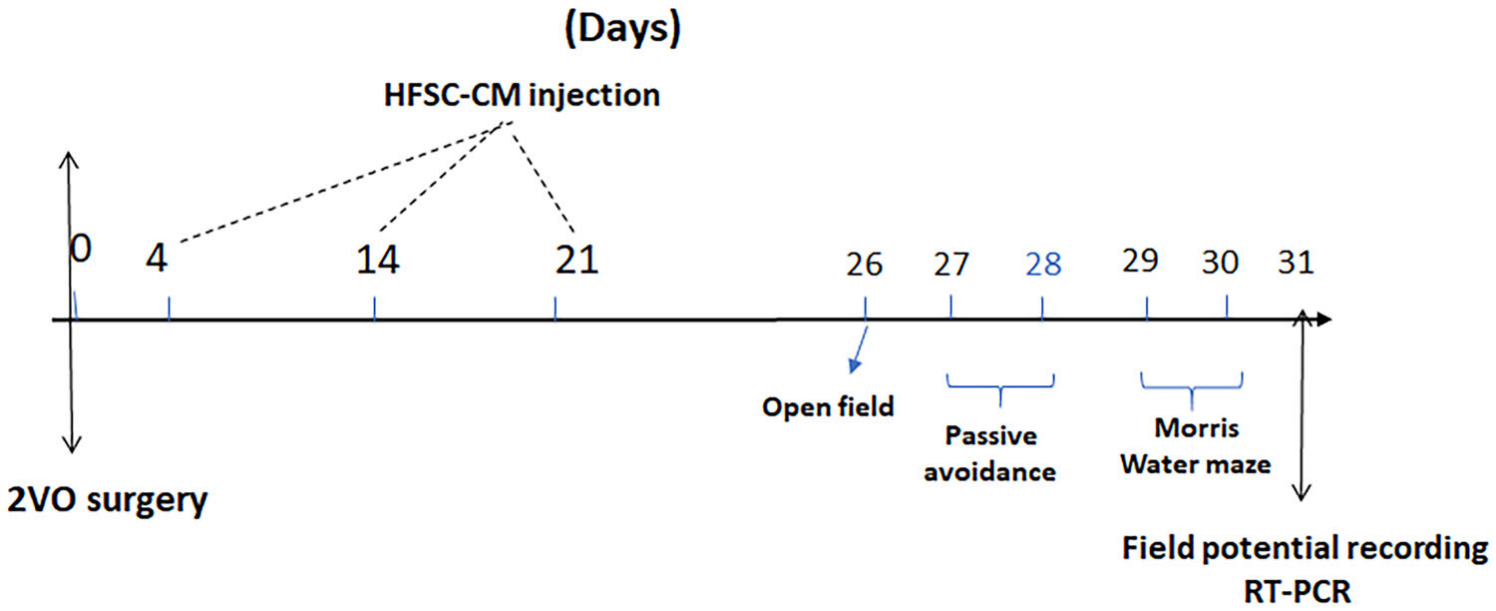
Timeline used to highlight the important methods in this study. HFSC‐CM, hair follicular stem cell‐conditioned medium.

All the methods used in this study were scheduled based on the following timeline (Figure 1). Animal studies were conducted under the protocols and guidelines approved by the Institutional Ethics Committee of Shiraz University of Medical Sciences (IR.SUMS.REC.1399.1179).

### 2.2 Induction of VaD through bilateral carotid occlusion (2VO)

VaD was induced using the 2VO model as previously described (Akbari et al., 2022). In this model, rats were anesthetized by the intraperitoneal (IP) injection of a mixture of ketamine (80 mg/kg) and xylazine (10 mg/kg) and placed on the heating blanket in the supine position. After shaving the anterior neck area, an incision was made on the ventral midline of the neck. By careful blunt dissection between the sternohyoid and the sternocleidomastoid muscles, the common carotid arteries were carefully explored. Then the small area of adventitial sheaths around the common carotid (without branches of nerves and blood vessels) was cut and freed to pass 4‐0 surgical silk and permanent ligation of the common carotid. At the end, the surgical area was sutured, and the animals were transferred to the animal cages and kept individually in separate cages at the animal house.

### 2.3 HFSC‐CM preparation

CM was prepared using HFSC obtained from rat whiskers. the details of this procedure and the verification of stem cells were described previously (Akbari et al., 2022).

The fourth passage of HFSC was used, and then the HFSC‐CM was established by seeding the HFSC per T75‐flask in a complete medium. Following reaching 80% of confluence, the cells were incubated for 48 h with serum‐free Alpha‐modified Eagle's minimum essential medium (α‐MEM) (BIO‐IDEA). After 48 h, CM was collected in a centrifuged tube, and to eliminate the large cell debris, CM was centrifuged at 1500 rpm for 5 min and then 3000 × *g* for 5 min at 4°C. Then, the HFSC‐CM was collected in a 50 mL falcon tube, and it was concentrated to 50‐fold of the original concentration by a freeze‐dryer method (Christ Alpha1‐2 LD Plus). The obtained lyophilized powder of HFSC‐CM was dissolved in PBS and quantified to measure the protein concentration by commercial Thermo Scientific bicinchoninic acid (BCA) Protein Assay Kit (BCA Protein Quantification Kit, Parstous, IR Iran) and α‐MEM was exploited in the control group. BCA assay was carried out following the manufacturer's guidance. The total protein of tested samples was calculated from a standard curve.

### 2.4 CM injection

In this study, we used the IP injection of lyophilized HFSC‐CM with a final protein concentration of 10 mg/mL in PBS for each animal on days 4, 14, and 24 after 2VO surgery (Figure 1). According to the previous studies, mesenchymal stem cell‐CM with protein concentration 10 mg has shown the protective effects on diabetes‐associated endothelial dysfunction (Yuan et al., 2016) and cognitive impairment (Aghaei et al., 2023).

### 2.5 Behavioral study

#### 2.5.1 Open‐field test

To assess animal anxiety levels, we used an OP apparatus on the 26th‐day post‐surgery. This apparatus measured 90 cm (length) × 90 cm (width) × 45 cm (height). Each animal was placed at the center of the test border and allowed to explore the environment for 15 min. The movements were recorded by a camera, and the results were analyzed using Ethovision software from Noldus Information Technology in the Netherlands. We recorded the number of grooming behavior and the time spent in in both peripheral and central regions. Before each new test, the device was thoroughly cleaned with 70% ethanol (Akbari et al., 2022).

#### 2.5.2 Passive‐avoidance test

To assess fear‐based conditioned avoidance learning and memory, we employed a PA test using a shuttle box apparatus. This box had two plexiglas chambers with two opposite colors white and black. Rats typically avoid bright lights and prefer the dark area. On the 27th‐day post‐surgery, each animal was initially placed in the white chamber and punished upon entrance to the black side by an electrical foot shock (0.5 mA, 50 Hz, 2 s once). This test was repeated with 5 min intervals. After the learning acquisition, the animal avoids going to the dark chamber. On the 28th‐day post‐surgery, the test was repeated and, the delay time before the entrance to the black chamber was recorded as step‐through latency (STL) for each animal (Shabani et al., 2009).

#### 2.5.3 Morris‐water maze (MWM)

The MWM test is a practical method for evaluating hippocampal spatial learning and memory (Vorhees & Williams, 2006). We used a black circular swimming pool suitable for rats with an escape platform in the target quadrant. On the 29th‐day post‐surgery, the rats underwent training with a visible escape platform (1.5 cm above the surface water), where they were required to swim for 60 s. Subsequently, the platform was submerged (1.5 cm below the surface water), and the memory acquisition was evaluated during three blocks. The time spent for each animal to find the hidden platform was recorded. Each block contains four trials. Twenty‐four hours after the last trial (on day 30th post‐surgery), the platform was removed to evaluate spatial memory tests (probe test). The percentage of time spent in the target quadrant was recorded as an index for spatial memory retention (Shabani et al., 2017).

### 2.6 Electrophysiological study

#### 2.6.1 Field potential recording

To investigate synaptic transmission and plasticity at CA3‐CA1 hippocampal excitatory synapses, we used field potential recording on day 31th post‐surgery. Each rat was anesthetized by the IP injection of urethane (1.5 g/kg), and tracheal cannulation was performed by neck incision. Subsequently, the animal was placed in the stereotaxic apparatus. The Schaffer collateral pathway is the axons of the neurons in the CA3 area that extend to the CA1 and form synapses in the CA1 area. By using the Paxinos atlas, the Schaffer collateral pathway and CA1 region were marked on the animal skull for the insertion of stimulating and recording electrodes, respectively, through the small holes that were drilled in these areas. After 25 min rest, evoked field excitatory postsynaptic potential (fEPSP) was recorded. Basal synaptic transmission (BST) was evaluated by the input/output I/O curve. To plot of I/O curve, the Schaffer collateral pathway was stimulated ranging from 50 to 1200 μA. The stimulus intensity for the 30 min baseline recording was set at 40% of the maximum amplitude responses in the I/O curve for each rat. High‐frequency stimulation (HFS) was induced at 80% of the maximum amplitude responses in the I/O curve, comprising 3 trains at 0.1 Hz, each consisting of 20 pulses at a frequency of 200 Hz. After HFS, the intensity of stimulation was resumed to 40% of the maximum response in the I/O curve and recording continued for 1 h. The level of long‐term potentiation (LTP) induction is the percentage change of fEPSP amplitude after HFS compared to the mean amplitude of the baseline recording.

### 2.7 Isolation of total RNA and real‐time PCR

The real‐time PCR method was used to quantify the expression levels of β1‐catenin, IGF‐1, TGF‐β, GSK‐3β, PSD‐95, and NR2B genes. For this purpose, the rat's hippocampus was quickly separated and frozen with liquid nitrogen and stored at −80°C. Subsequently, RNA extraction was carried out using a specialized kit (RNX plus solution, Sinaclon, IR Iran), the extraction process was performed according to the kit protocol. The extracted RNA was converted into cDNA through the kit protocol (Easy cDNA Synthesis Kit, Parstous, IR Iran) which was used in the real‐time PCR (StepOnePlus Real‐Time PCR system) by low‐rox master mix (Ampliqon). The primers used are detailed in Table 1.

**TABLE 1:** Primer sequences (5′–3′) used in qPCR.

Gene name (ID)	Forward primer (F1) (5′ to 3′) Reverse primer (R1) (5′ to 3′)	Ta(c)	Product length (BP)	Exons
IGF1 (Insulin growth factor Gene ID: 24482	F1‐TGGTGGACGCTCTTCAGTTC R1‐TCCGGAAGCAACACTCATCC	57–61	123	2–3
Gsk‐3β (glycogen synthase kinase 3 beta) Gene ID: 84027	F1‐AGCTGATCTTTGGAGCCACC R1‐CTGATCCACACCACTGTCCC	56–61	119	6–7
TGF‐β1 (transforming growth factor, beta 1) Gene ID: 59086	F1‐TGACATGAACCGACCCTTCC R1‐TGCCGTACACAGCAGTTCTT	57–62	138	5–6
PSD95 (discs large MAGUK scaffold protein 4) Gene ID: 29495	F1‐CTGCATCCTTGCGAAGCAAC R1‐AAGAAACCGCAGTCCTTGGT	57–62	85	11–12
NR2B (glutamate ionotropic receptor NMDA type subunit 2B) Gene ID: 24410	F1‐GGTTTCTGGCCTGAGTGACA R1‐TCCTCTCTGTGCTGCCATTG	57–61	95	9–10
β1‐Catenin(catenin beta 1) Gene ID: 84353	F1‐GAGCACATCAGGACACCCAG R1‐CTATCTCCTCCATGCGGACG	57–61	84	9–10
GAPDH (glyceraldehyde‐3‐phosphate dehydrogenase) Gene ID: 24383	F1‐AGTGCCAGCCTCGTCTCATA R1‐GAGAAGGCAGCCCTGGTAAC	57–61	91	1–2–3

### 2.8 Statistical analysis

Data management and analysis were performed using Prism 7 and SPSS 20 software. We performed a power analysis to determine the appropriate sample size for our study. All data were expressed as mean ± SEM. At first, the normal distribution of data was assessed by the Kolmogorov–Smirnov test. Kruskal–Wallis with Dunn's post hoc test or one‐way ANOVA with Tukey's post hoc test was used to compare the results of behavioral tests, gene expression (after calculating Δ*Ct* and mean Δ*Ct* ± SE), I/O curve, and LTP induction. To evaluate alteration in fEPSP amplitudes before and after HFS at different times two‐way ANOVA, with repeated measures was used. A *p*‐value <.05 was statistically significant in all tests.

## 3 RESULTS

The results from this study indicated no statistical difference in any of the parameters between the sham and control groups.

### 3.1 Behavioral tests

#### 3.1.1 Open‐field test

**FIGURE 2:**
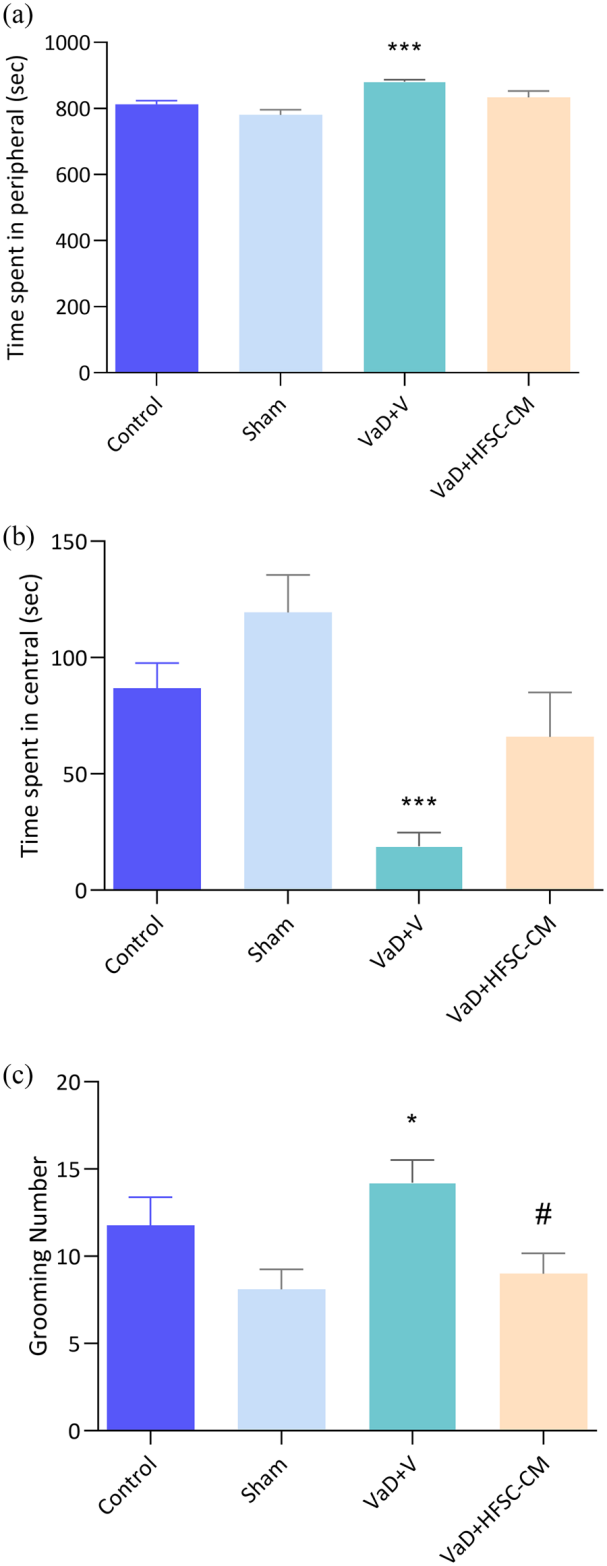
The open‐field test. The peripheral time (a), the central time (b), and grooming number (c) in different studied groups. In the treatment group, grooming number was recovered compared to vascular dementia (VaD) + V. The values are shown as mean ± SEM. Significant differences with respect to the sham *p < .05, ***p < .001) and VaD + V (#*p* < .05), control (*n* = 9), sham‐operation (sham, *n* = 10), VaD + V (*n* = 9), and VaD + hair follicular stem cell (HFSC)‐conditioned medium (CM) (*n* = 12). One‐way ANOVA with the Tukey's post hoc test.

In this behavioral test, it is evident that anxious animals spent more time in the peripheral zone than in the central zone, displaying a higher number of grooming behaviors compared to non‐anxious animals. As shown in Figure 2a,b, in the VaD + V group, the time spent in the peripheral significantly increases relative to the sham (881 ± 5.7 s vs. 780.6 ± 16 s; *p* < .001), whereas the central time significantly decreases compared to the sham groups (18.9 ± 5.7 s vs. 119.5 ± 16 s; *p* < .001). Treatment with HFSC‐CM did not show significant recovery in peripheral and central times in the VaD + HFSC‐CM group relative to the VaD + V group, but this group also did not show any difference compared to the sham group. Additionally, there was a significant increase in the grooming number of the VaD + V group compared to the sham group (14.2 ± 1.3 vs. 8 ± 1.1; *p <* .05), and injection of HFSC‐CM led to a decrease in grooming number in the VaD + HFSC‐CM group relative to VaD + V (9 ± 1.1 vs. 14.2 ± 1.3; *p* < .05) (Figure 2c) [(*F* (3, 36) = 4.402, *p* = .0097].

#### 3.1.2 Passive‐avoidance test

**FIGURE 3:**
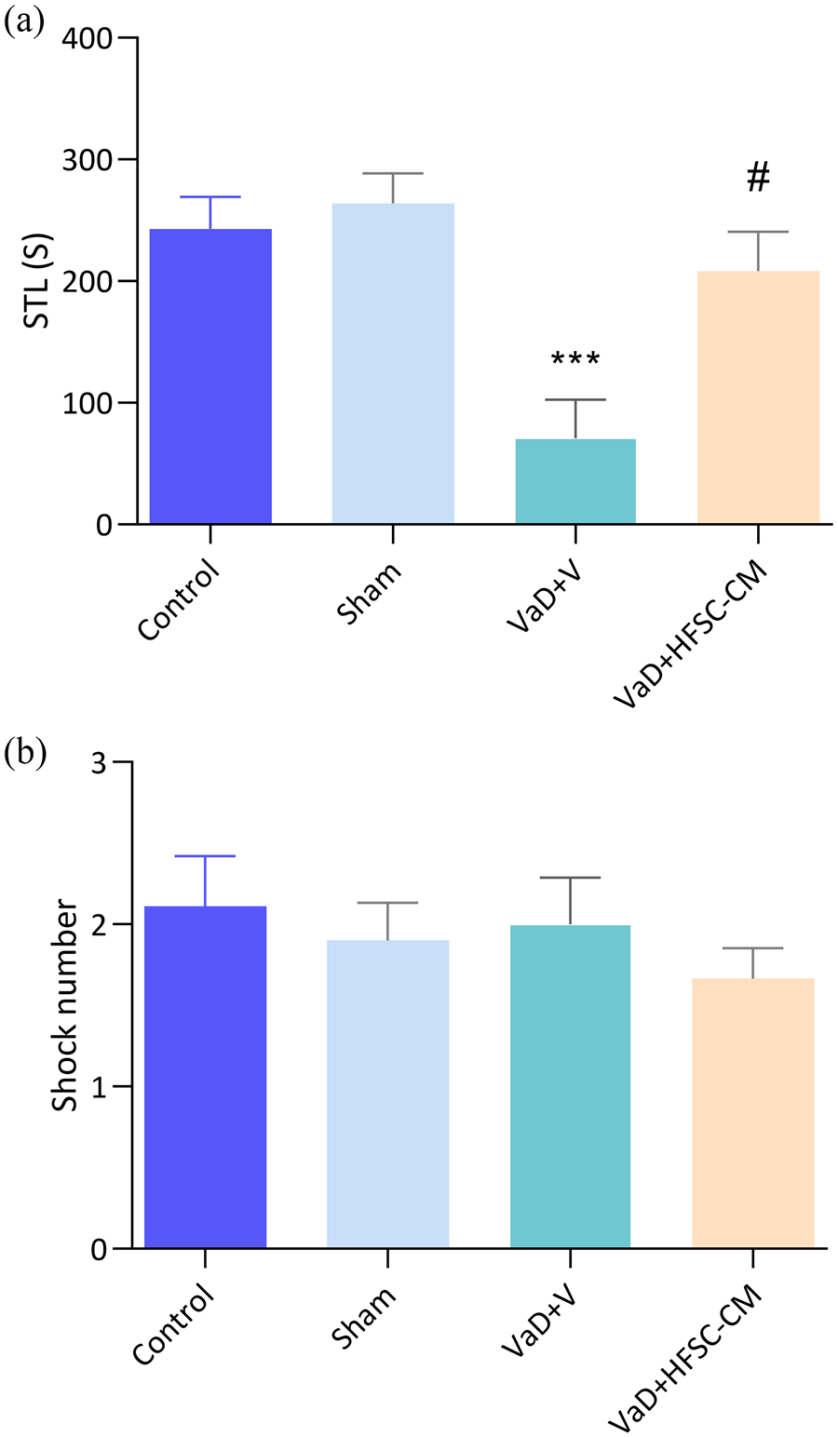
The passive‐avoidance test. The step‐through latency (STL) (a) and shock number (b) in the studied groups. The values are shown as mean ± SEM. Significant differences with respect to the sham (***p < .001) and vascular dementia (VaD) + V (#*p* < .05), control (*n* = 9), sham‐operation (sham, *n* = 10), VaD + V (*n* = 9), and VaD + hair follicular stem cell (HFSC)‐conditioned medium (CM) (*n* = 12). One‐way ANOVA with the Tukey's post hoc test.

The fear memory impairment is evident through STL time depression. As shown in Figure 3a, the STL time significantly decreased in the VaD + V group compared to the sham (70.7 ± 31.7 s vs. 263.8 ± 24.6 s) (*p <* .001). However, the injection of HFSC‐CM in VaD rats increased the STL compared to the VaD + V group (208.1 ± 32.2 s vs. 70.7 ± 31.7 s) (*p <* .05), [F (3, 36) = 7.939, *p* = .0003]. Notably, there was no significant difference in the shock number between experimental groups (Figure 3b).

#### 3.1.3 Morris‐water maze test

**FIGURE 4:**
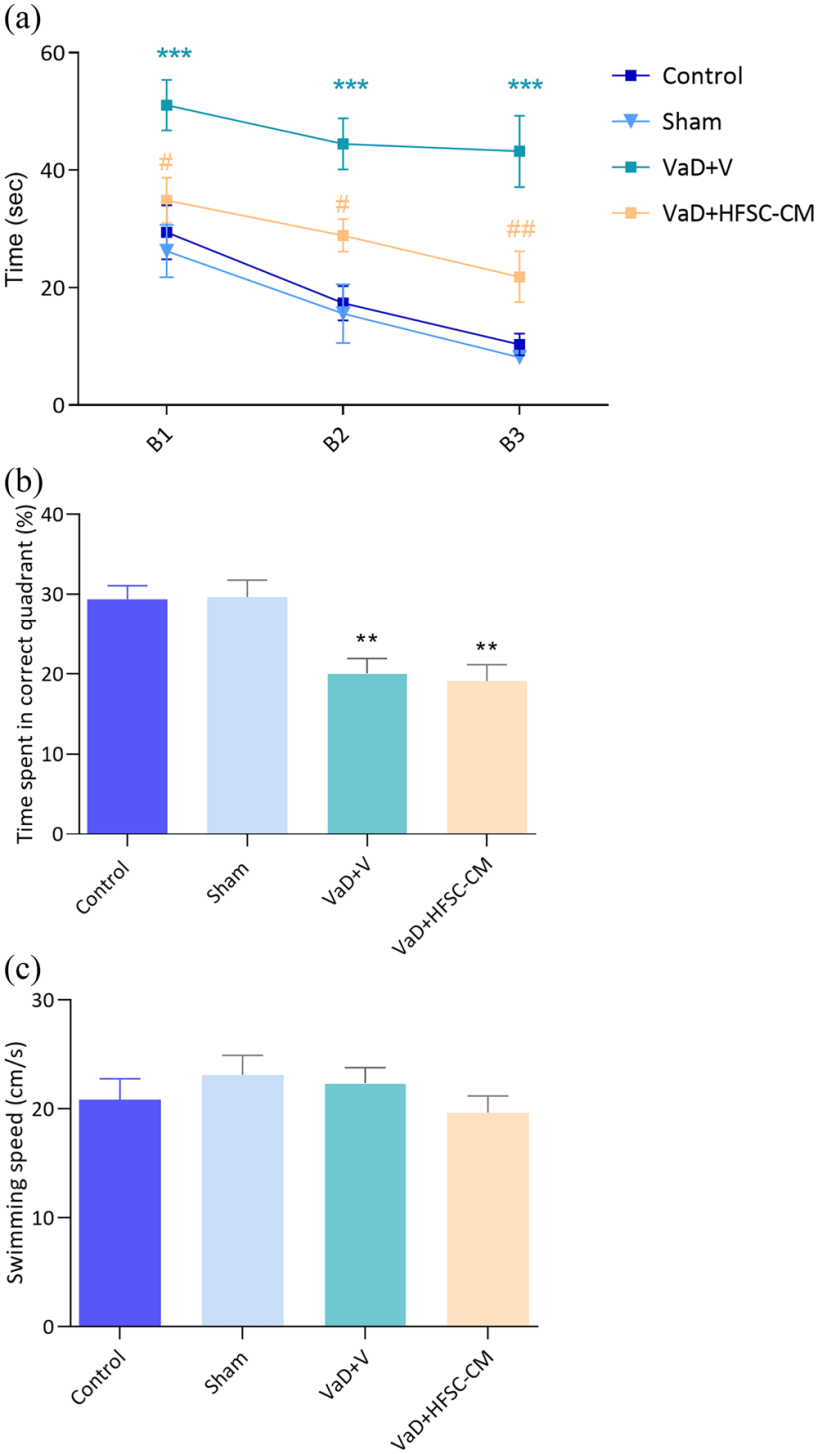
The Morris‐water maze test. The learning trials during three blocks (B1, B2, B3) in different groups (two‐way ANOVA) (a), spatial memory retention was evaluated by probe test (b), swimming speed in all groups (c). The values are shown as mean ± SEM. Significant differences with respect to the sham (**p < .01, ***p < .001) and vascular dementia (VaD) + V (#*p* < .05, ##*p* < .01), control (*n* = 9), sham‐operation (sham, *n* = 10), VaD + V (*n* = 9), and VaD + hair follicular stem cell (HFSC)‐conditioned medium (CM) (*n* = 12). One‐way ANOVA with the post‐hoc test.

In this test, using the spatial cues around the maze, animals learn the location of the hidden escape platform in the target quadrant during 12 trials. Initially, the escape platform is visible, then it is hidden, and finally to evaluate the memory, the platform is removed. We considered the escape latency (time spent to find the platform) as an index for spatial learning‐memory during three blocks (each block contains four trials). During the 3 blocks (B1, B2, and B3) VaD + V rats showed a significant increase in escape latency compared to the sham group (*p <* .001) (Figure 4a). However, the escape latency time significantly decreased following treatment with HFSC‐CM in three blocks: (B1; 34.8 ± 3.8 s vs. 51 ± 4.2, *p <* .05), (B2; 28.8 ± 2.7 s vs. 44.9 ± 4.3, *p* < .05), and (B3; 21.8 ± 4.3 s vs. 43.2 ± 6, *p <* .01). In the probe trial, where the escape platform was removed from the target quadrant, the percentage of swimming time in this quadrant was calculated (Figure 4b). Rats with VaD exhibited a significant reduction in this percentage compared to the sham group (20.1% ± 1.8% vs. 29.6% ± 2.1%; *p <* .01), indicating spatial memory disturbance. There was no significant recovery in spatial memory retention following HFSC‐CM injection (*p* > .05) [F (3, 35) = 8.490, *p* = .0002]. Importantly, animals in all groups swam at the same speed (Figure 4c).

### 3.2 Field potential recording

#### 3.2.1 Basal synaptic transmission (BST)

**FIGURE 5:**
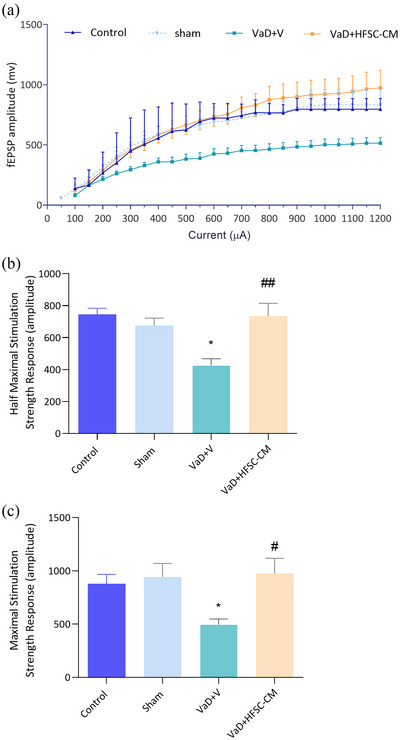
The basal synaptic transmission (BST) of CA1 neurons was assessed through the input/output curve (a). The half (b) and maximum (c) stimulation field excitatory postsynaptic potential (fEPSP) amplitude. Significant differences with respect to the sham (*p < .05) and vascular dementia (VaD) + V (#*p* < .05, ##*p* < .01). The values are shown as mean ± SEM. Significant differences with respect to the sham (*p < .05) and VaD + V (#*p* < .05, ##*p* < .01), control (*n* = 9), sham‐operation (sham, *n* = 10), VaD + V (*n* = 9), and VaD + hair follicular stem cell (HFSC)‐conditioned medium (CM) (*n* = 10). One‐way ANOVA with the Tukey's post hoc test.

A significant right and downward shift in the I/O curve indicates a decline in BST. As shown in Figure 5a, the VaD model could decrease the BST in the VaD + V group relative to the sham group.

The amplitude of fEPSP in half‐maximal and maximal stimulation response significantly decreased in the VaD + V group compared to the sham group (one‐way ANOVA analysis) (half max; 426.1 ± 42 vs. 690.6 ± 49.4; *p* < .05) (max; 514.3 ± 46.1 vs. 943.6 ± 125.6; *p* < .05). We found a significant increase in the half‐maximum response following HFSC‐CM injection compared to the VaD + V group (734.7 ± 79.8 vs. 426.1 ± 42; *p* < .01) [*F* (3, 34) = 6.912, *p* = .0009] (Figure 5b). Furthermore, treatment with HFSC‐CM significantly increases the maximal stimulation response (974.0 ± 143.8 vs. 514.3 ± 46.1; *p* < .05) [*F* (3, 34) = 3.837, *p* = .0181] with respect to VaD + V group (Figure 5c).

#### 3.2.2 Long‐term synaptic plasticity (LTP)

**FIGURE 6:**
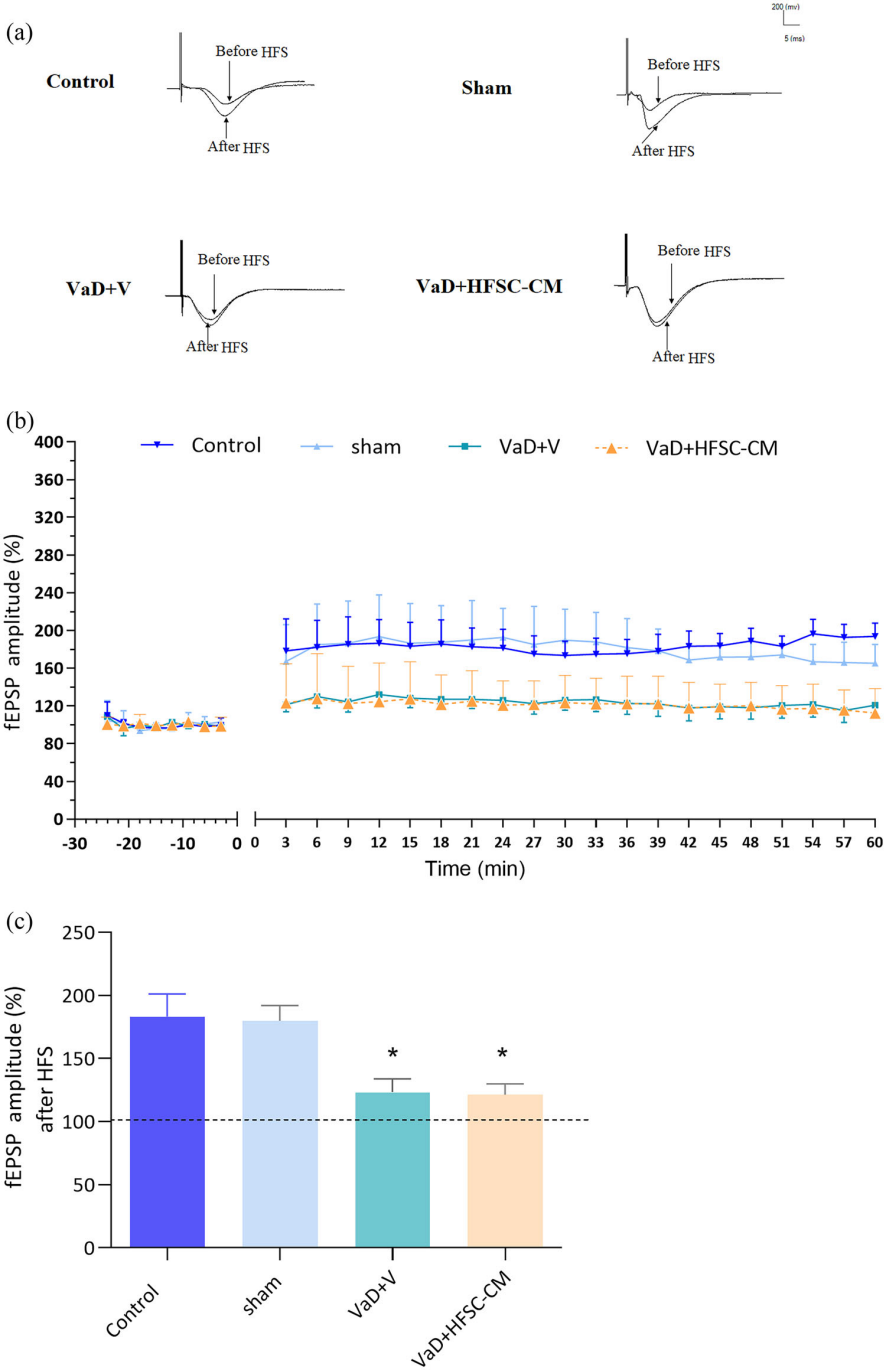
The long‐term synaptic plasticity (LTP) was recorded from CA1 neurons after high‐frequency stimulation (HFS). The sample traces of responses (a), the baseline and LTP recording (b), and the means of field excitatory postsynaptic potential (fEPSP) amplitude after HFS (c). The values are shown as mean ± SEM. Significant differences with respect to the sham (*p < .05), control (*n* = 9), sham‐operation (sham, *n* = 10), vascular dementia (VaD) + V (*n* = 9), and VaD + hair follicular stem cell (HFSC)‐conditioned medium (CM) (*n* = 10). One‐way ANOVA with the Tukey's post hoc test.

The sample traces of responses (Figure 6a) and LTP curves (Figure 6b) in experimental groups showed that LTP induction was impaired in VaD + V rats, and there was no significant difference in LTP induction between treated and VaD groups.

As shown in Figure 6c, the mean of the post‐HFS fEPSP amplitude was compared between groups. One‐way ANOVA analysis showed that the percentage of LTP induction significantly decreased in the VaD + V group compared to the sham group (123.5% ± 10.2% vs. 179.9% ± 12%; *p* < .05). HFSC‐CM treatment could not recover the percentage of LTP induction relative to the VaD + V group (121.3% ± 8.6% vs. 123.5% ± 10.2%; *p* > .05).

### 3.3 mRNA expression levels of β1‐Catenin, IGF‐1, TGF‐β, GSK‐3β, PSD‐95, and NR2B in rat hippocampus

**FIGURE 7:**
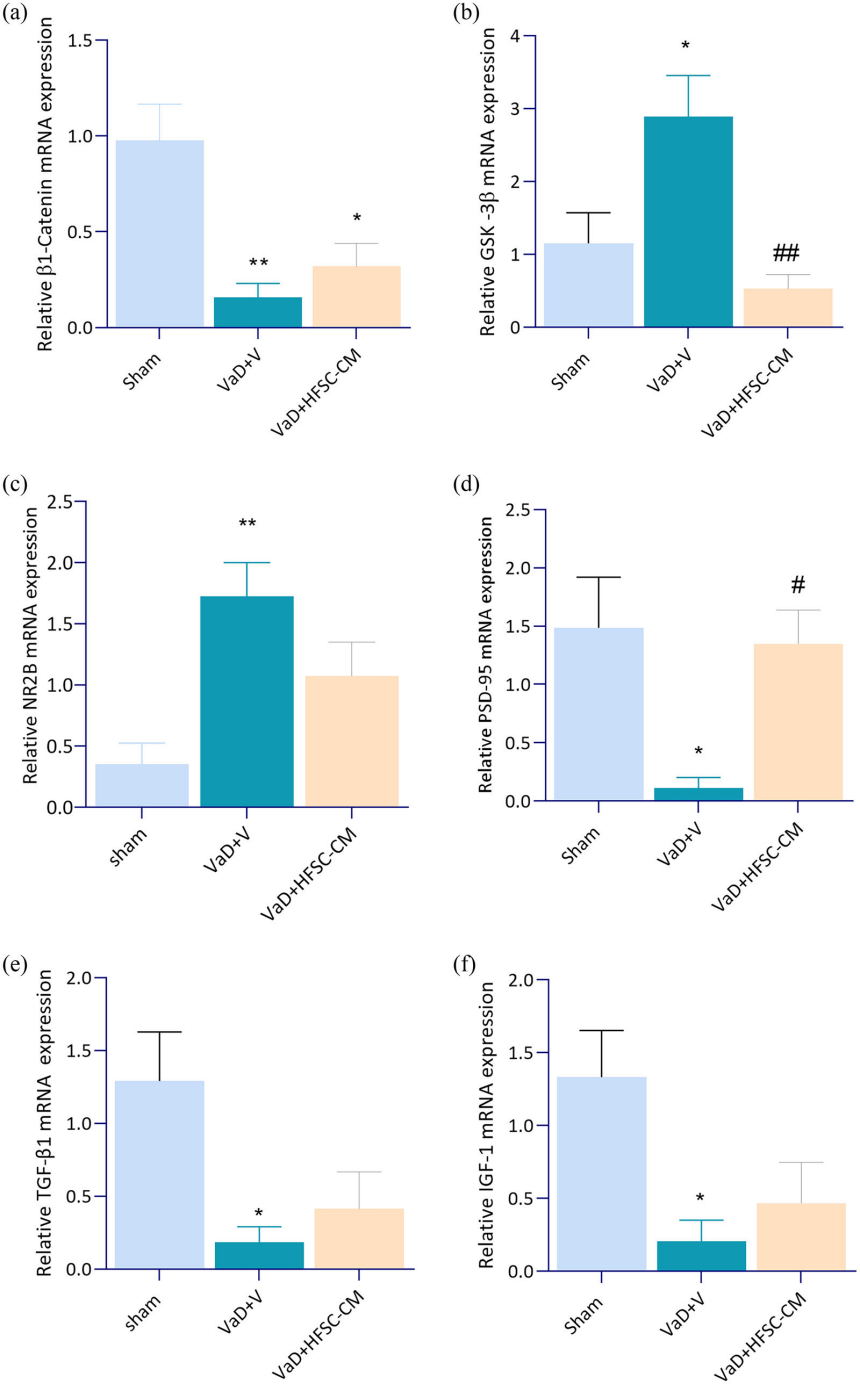
The mRNA expression of β1‐catenin (a), glycogen synthase kinase‐3β (GSK‐3β) (b), NR2B (c), postsynaptic density protein 95 (PSD‐95) (d), transforming growth factor‐beta (TGF‐β) (e), and insulin‐like growth factor‐1 (IGF‐1) (f), in rat hippocampus. Significant differences with respect to the sham (*p < .05, ** *p* < .01) and vascular dementia (VaD) + V (#*p* < .05, ##*p* < .01). The values are shown as mean ± SEM. sham‐operation (sham, *n* = 7), VaD + V (*n* = 6), and VaD + hair follicular stem cell (HFSC)‐conditioned medium (CM) (*n* = 7). One‐way ANOVA with the Tukey's post hoc test.

In the hippocampus of VaD rat, mRNA expression levels of β1‐Catenin, IGF‐1, TGF‐β, and PSD‐95 significantly decreased (*p* < .05), whereas mRNA expression of GSK‐3β and NR2B significantly increased compared with the sham group (*p* < .05). Following treatment with HFSC‐CM, mRNA expression level of PSD‐95 and GSK‐3β significantly recovered in VaD + HFSC‐CM group relative to VaD + V (PSD‐95; 1.34 ± 0.28 vs. .11 ± 0.09; *p* < .05) (GSK‐3β; .52 ± .19 vs. 2.89 ± 0.56; *p* < .01), [*F* (2, 17) = 8.773, *p* = .0024] (Figure 7a–c).

## 4 DISCUSSION

The study results suggest the efficacy of HFSC‐CM therapy in 2VO‐induced VaD. HFSC‐CM administration effectively improved impaired fear memory and spatial learning in 2VO rats. Furthermore, the injection of 10 mg of HFSC‐CM on days 4, 14, and 24 after 2VO rescued attenuation in the BST, although there was no considerable positive impact in LTP induction relative to 2VO rats. Additionally, the treated group showed the overexpression of PSD‐95 as a synaptic marker and GSK‐3β downregulation compared to the VaD + V group, accompanied by an improvement in hippocampal BST. We hypothesized that repeated HFSC‐CM administration might enhance the synaptic transmission and plasticity in the CA1 hippocampus of 2VO rats. The present findings appear consistent with other research which found a decrease in hippocampal mRNA expression of β1‐Catenin (Bahlakeh et al., 2022), PSD‐95 (Shao et al., 2011), IGF‐1 (Gong et al., 2012), and TGF‐β1 (Caraci et al., 2012) as well as an increase in expression of GSK‐3β (Gomez‐Sintes et al., 2011), and NR2B (Liu et al., 2012) following dementia models. Similarly, impairment in different behavioral tests such as OP, PA, and MWM in 2VO rats is consistent with the findings of other studies, which reported CCH‐induced learning memory impairment and anxiety‐like behavior (Fu et al., 2014; Venkat et al., 2015).

The structural and functional changes in hippocampal CA1 synapse are major causes of BST and LTP impairments in neurodegenerative dementia (Dong et al., 2015; Weerasinghe‐Mudiyanselage et al., 2022). In a previous study, we demonstrated that the transplantation of HFSC had a promising effect in the LTP recovery following the VaD model, without a significant improvement in BST (Akbari et al., 2022). However, in the present study, we found a potential protective effect of HFSC‐CM in the BST recovery in the treated group, whereas there was no significant improvement in LTP induction. Moreover, the repeated injection of 10 mg HFSC‐CM on days 4, 14, and 24 after 2VO surgery restored PSD‐95 and GSK‐3β levels in 2VO rats.

Changes in synaptic transmission strength play a crucial role in processes related to the storage and retrieval of information (Kennedy, 2013). The processes of synaptic transmission are tightly modulated through, both presynaptic and postsynaptic mechanisms (Casillas‐Espinosa et al., 2012). Presynaptic nerve terminals control the synaptic transmission via Ca^2+^ signaling, neurotransmitter release, formation, and recycling of synaptic vesicles (Mochida, 2020). Postsynaptic factors, including the postsynaptic density, postsynaptic receptors, and proteins, modulation of signaling cascades, and signal communication, also play vital roles in synaptic transmission (Madrigal et al., 2019). PSD‐95 is a key synaptic scaffolding molecule that supports the postsynaptic protein complex at the synaptic contact zone, organizing the postsynaptic receptors and signal transduction molecules (Dosemeci et al., 2007). The overexpression of PSD‐95 can protect synapses from Aβ toxicity (Dore et al., 2021). Numerous studies have highlighted the role of PSD‐95 in synaptic transmission and plasticity (Dore & Malinow, 2021; Juan et al., 2014; Tai et al., 2022; Xu, 2011). The association of PSD‐95 and neurodegenerative disorders provides promising clues for future therapeutic approaches (Levy et al., 2022; Ugalde‐Trivino & Diaz‐Guerra, 2021). The evidence from previous studies has suggested that PSD‐95 was upregulated following treatment with human HFSC‐CM in the ipsilateral hippocampal stroke model (Karimi‐Haghighi et al., 2023). There have been several longitudinal in vitro and in vivo studies that have reported the protective effects of stem cell‐derived extracellular vesicles on the preservation of PSD‐95 expression (Cui et al., 2018; de Godoy et al., 2018). El‐Husseini et al. (2000) published a paper in which they described that the PSD‐95 overexpression in post‐synapses of hippocampal neurons has multiple effects including; maturation of glutamatergic synapses, enhanced postsynaptic clustering, the upregulation of AMPAR, increase in presynaptic release probability, increased the number and size of dendritic spines, and increases in AMPAR containing synapses. Other researchers have reported the association of PSD‐95 with synaptic strength (Zhang & Lisman, 2012) and survival and maintenance of the spine (Cane et al., 2014). Moreover, dephosphorylation ser 9 of GSK‐3β results in the activation of the enzyme. Active GSK‐3β can phosphorylate a wide range of substrates including several different metabolic enzymes, transcription factors, and proteins that are involved in cell division and cell adhesion (Frame & Cohen, 2001). Previous research has indicated that the possible therapeutic benefits of GSK‐3 antagonists for neurodegenerative models such as Alzheimer's (Selenica et al., 2007), amyotrophic lateral sclerosis (Koh et al., 2007), spinocerebellar ataxia type 1 (Watase et al., 2007), Huntington's disease (Wood & Morton, 2003), and hippocampal epileptic neurodegeneration (Busceti et al., 2007). The role of GSK‐3β signaling has been identified in hippocampal synaptic transmission and plasticity (Peineau et al., 2008).

The conditioned media from stem cells serves as a rich source of growth factors, contributing to the activation of endogenous repair mechanisms. This process involves the mobilization of endogenous stem cells (Osugi et al., 2012). Additionally, it plays a role in dendritic remodeling, maturation of spines, and the promotion of synaptogenesis (Asgari Taei et al., 2022; Lin et al., 2023). In view of all that has been mentioned so far, one may suppose that treatment with HFSC‐CM through PSD‐95 overexpression and GSK‐3β downregulation plays a major role in the BST improvement in our treated group. Considering the well‐known significant roles of PSD‐95 and GSK‐3 in synaptic transmission (Amici et al., 2021; Keith & El‐Husseini, 2008), hippocampal neurogenesis (Kisoh et al., 2017; Morales‐Garcia et al., 2012; Wi et al., 2016), and synaptogenesis (Nikonenko et al., 2008), it seems possible that BST improvement in our results is due to the direct neurotrophic effects of HFSC‐CM on synaptic transmission and/or indirectly through the enhancement of hippocampal neurogenesis and increase in new spines. As described in our previous study, the transplantation of one million HFSC on days 4, 14, and 24 after 2VO significantly recovered LTP induction in 2VO rats (Akbari et al., 2022). Despite this, repeated injection of 10 mg of HFSC‐CM after 2VO failed to restore LTP induction and spatial memory. A possible explanation for these results is likely to be attributable to the lack of adequate HFSC‐CM in the rat's brain. Therefore, we suggest that to achieve more effectiveness of CM on LTP induction and spatial memory improvement, an additional dose of CM may be needed in 2VO rats.

The behavioral improvements observed in the HFSC‐CM treated group, especially in fear memory, spatial learning, and grooming, add another layer of complexity to the intricate relationship among memory, synaptic processes, and the potential therapeutic effects of stem cell‐derived factors. Interestingly, in the treated group, the recovery of fear memory and learning is associated with the impairment of LTP induction recovery. The MWM test is usually used to evaluate hippocampal‐dependent learning memory (Cazakoff et al., 2010), and LTP at CA3–CA1 hippocampal synapse is indeed a main cellular mechanism for memory storage (Kumar, 2011). However, the PA test is widely used to assess fear learning and memory in rodents, and the anterior portion of the basolateral nucleus (BLA) of the amygdala is the main structure in the formation of fear memory (Gale et al., 2004). Furthermore, LTP in the amygdala is associated with fear conditioning, and BLA stimulation can facilitate the dentate gyrus LTP (Ikegaya et al., 1996; Sah et al., 2008). Although LTP at CA3–CA1 synapse has a critical role in the acquisition of associative learning (Gruart et al., 2006), it has been observed that sometimes, despite a deficit in LTP induction, contextual fear memory formation can still be formed, and it was associated with the generation of multi‐innervated dendritic spines (Aziz et al., 2019; Giese et al., 2015).

In addition, the improvement in synaptic transmission observed with HFSC‐CM therapy adds a crucial electro‐physiological dimension to its potential therapeutic effects. Numerous studies have attempted to explain the enhancement of BST through an increase in multi‐innervated dendritic spines (Aziz et al., 2019; Tonnesen & Nagerl, 2016). In aged mice, the improvement in hippocampal memory storage is associated with multi‐input synapses, but not with LTP‐strengthened synapses (Aziz et al., 2019). The current study found a significant decrease in grooming behavior following HFSC‐CM therapy. A strong relationship between anxiety‐like behavior and stress with endogenous neurogenesis has been reported in prior studies (Schoenfeld & Gould, 2012). Another possible explanation for this is improved decision‐making (Sestakova et al., 2013). A person's ability to make decisions is impaired in people with dementia because the disease affects the parts of the brain such as temporal, frontal, and parietal regions, and these areas are associated with decision‐making and vulnerable to age‐related change (Sun et al., 2021; van Duinkerken et al., 2018).

This combination of findings, especially the enhancement in fear memory and decision‐making, provides some support for the conceptual premise that HFSC‐CM therapy has a potential for improving synaptic transmission in the wide areas of the brain beyond the hippocampus. The most obvious finding to emerge from this study is BST improvement in CA3‐CA1 synapse, which was accompanied by improvement in spatial learning and fear memory as well as anxiety decline. Recovered hippocampal mRNA expression of PSD‐95 and GSK‐3β was the second major finding that could confirm the improvement of synaptic transmission. Further research should be done to investigate the beneficial effects of HFSC‐CM therapy in the VaD model with higher doses than 10 mg or with repeated doses of more than three. This makes it possible to observe the LTP induction in the treated group. Moreover, hippocampal proteins level of PSD‐95 and GSK‐3β as well as endogenous neurogenesis markers should be evaluated following injection of HFSC‐CM. Taken together, considering the pivotal role of synaptic transmission in dementia the BST enhancement in CA3‐CA1 synapse of VaD rats with HFSC‐CM therapy was the most important outcome of this study which provides a possible implication for the therapeutic potential of using HFSC‐CM to treat dementia.

## AUTHOR CONTRIBUTIONS

Mojtaba Ghobadi and Somayeh Akbari planed the methodology for the result and analysis, drafted the article, and revised it critically for important intellectual content and approved the version to be published. Mahnaz Bayat, Seyed Mostafa Shid Moosavi, and Masoud Haghani have made a substantial contribution to the concept and design of the article, analysis and interpretation of data for the article, revised it critically for important intellectual content. Mohammad Saied Salehi, Sareh Pandamooz, Negar Azarpira, Afsoon Afshari, and Etrat Hooshmandi planed the methodology for the result, interpretation of data for the article, drafted the article, and revised it critically for important intellectual content and approved the version to be published.

### PEER REVIEW

The peer review history for this article is available at https://publons.com/publon/10.1002/brb3.3351.

## Data Availability

Data available on request.
